# The generation of oligodendroglial cells is preserved in the rostral migratory stream during aging

**DOI:** 10.3389/fncel.2013.00147

**Published:** 2013-09-11

**Authors:** Vivian Capilla-Gonzalez, Arantxa Cebrian-Silla, Hugo Guerrero-Cazares, Jose M. Garcia-Verdugo, Alfredo Quiñones-Hinojosa

**Affiliations:** ^1^Brain Tumor Stem Cell Laboratory, Department of Neurosurgery, Johns Hopkins School of MedicineBaltimore, MD, USA; ^2^Laboratory of Comparative Neurobiology, Instituto Cavanilles de Biodiversidad y Biologia Evolutiva, University of Valencia, CIBERNEDPaterna, Valencia, Spain

**Keywords:** neuroblast migration, subventricular zone, rostral migratory stream, olfactory bulb, neurogenesis, oligodendrogenesis, aging

## Abstract

The subventricular zone (SVZ) is the largest source of newly generated cells in the adult mammalian brain. SVZ-derived neuroblasts migrate via the rostral migratory stream (RMS) to the olfactory bulb (OB), where they differentiate into mature neurons. Additionally, a small proportion of SVZ-derived cells contribute to the generation of myelinating oligodendrocytes. The production of new cells in the SVZ decreases during aging, affecting the incorporation of new neurons into the OB. However, the age-related changes that occur across the RMS are not fully understood. In this study we evaluate how aging affects the cellular organization of migrating neuroblast chains, the proliferation, and the fate of the newly generated cells in the SVZ-OB system. By using electron microscopy and immunostaining, we found that the RMS path becomes discontinuous and its cytoarchitecture is disorganized in aged mice (24-month-old mice). Subsequently, OB neurogenesis was impaired in the aged brain while the production of oligodendrocytes was not compromised. These findings provide new insight into oligodendrocyte preservation throughout life. Further exploration of this matter could help the development of new strategies to prevent neurological disorders associated with senescence.

## Introduction

Neurogenesis persists in the subventricular zone (SVZ) of the lateral ventricles throughout life. SVZ astrocytes continuously proliferate and give rise to intermediate progenitor cells, which then differentiate into neuroblasts forming large chains ensheathed by gliotubes of astrocytes (Doetsch et al., 1997; Ponti et al., 2013). These migratory structures emerge from the SVZ and coalesce into the rostral migratory stream (RMS) that ends in the olfactory bulb (OB) (Lois et al., 1996; Peretto et al., 1997; Alvarez-Buylla and Garcia-Verdugo, 2002). Thus, neuroblasts migrate tangentially from the SVZ and incorporate into the OB. Neuroblasts in the RMS retain their ability to proliferate (Smith and Luskin, 1998; Poon et al., 2010), but once they reach the OB, neuroblasts begin radial migration and mature into interneurons that integrate in preexisting functional circuits (Lois and Alvarez-Buylla, 1994; Luskin et al., 1997; Carleton et al., 2003; Imayoshi et al., 2008; Kelsch et al., 2010; Lazarini and Lledo, 2011). Although most SVZ precursor cells generate neuroblasts to support OB neurogenesis, a small subpopulation of precursors gives rise to cells in the oligodendroglial lineage, which are able to migrate toward the corpus callosum, striatum, or septum to differentiate into myelinating oligodendrocytes (Nait-Oumesmar et al., 1999; Menn et al., 2006; Gonzalez-Perez et al., 2009; Gonzalez-Perez and Alvarez-Buylla, 2011; Capilla-Gonzalez et al., in press).

During aging, the proliferative potential of the rodent SVZ decreases due to a partial depletion of neural precursor cells, with a subsequent disruption of OB neurogenesis (Maslov et al., 2004; Luo et al., 2006; Molofsky et al., 2006; Bouab et al., 2011; Conover and Shook, 2011; McGinn et al., 2012). Similarly, the human SVZ maintains its proliferative and neurogenic potential in adult life but it is drastically decreased when compared to fetal and pediatric stages (Sanai et al., 2004; Quinones-Hinojosa et al., 2006; Guerrero-Cazares et al., 2011; Sanai et al., 2011). However, the effects of aging on the genesis of new cells have mostly been studied at the SVZ level with little emphasis on the migratory pathway that the SVZ-derived cells follow to reach the OB (Bouab et al., 2011; Shook et al., 2012). Here, we investigate the age-related changes occurring across the RMS of mice. We demonstrate that this migratory pathway is deeply altered during aging and tends to disappear, resulting in a disruption of the OB neurogenesis. Interestingly, we found that the production of oligodendrocytes in the SVZ-OB system is not compromised by aging. Our study provides a better interpretation of the neurogenesis decline occurring in the aged brain, which could help to understand neurological disorders associated with senescence.

## Results

### The RMS cytoarchitecture is disrupted by aging

**Figure 1:**
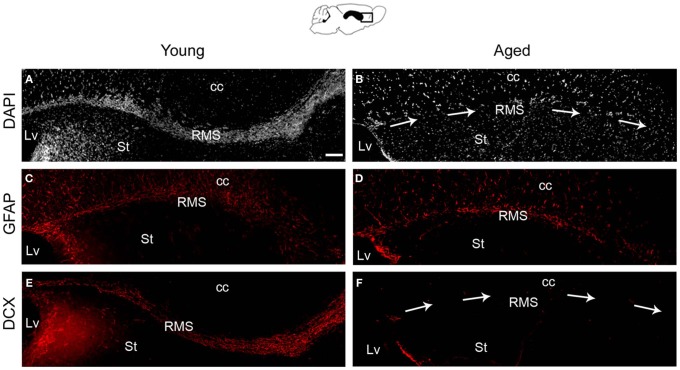
**The population of neuroblasts within the RMS is disrupted during aging.** Sagittal sections of the RMS in young and aged mice. **(A)** Young RMS stained with DAPI. **(B)** Aged RMS stained with DAPI, showing a notable reduction of cells in the migratory pathway (arrows). **(C)** Young RMS immunolabeled with GFAP. **(D)** Aged RMS immunolabeled with GFAP did not reveal remarkable differences in the population of GFAP+ cells. **(E)** Young RMS immunolabeled with DCX. **(F)** Aged RMS (arrows) immunolabeled with DCX, showing a notable reduction of neuroblasts. cc, corpus callosum; St, striatum; Lv, lateral ventricle. Scale bar: 100 μm

Consistent with previous studies (Luo et al., 2006; Bouab et al., 2011), the SVZ of mice showed a loss of cells during aging, particularly in the population of neuroblasts forming migratory chains (Figure S1). To evaluate if those effects from aging were also present in the RMS, sagittal sections of the brain were first stained with DAPI nuclear dye. We observed that the population of cells that form the RMS connecting the SVZ with the OB was diminished in the aged brain (Figures 1A,B and Figure S2). The astrocytic and neuroblast populations within the RMS were examined using the glial fibrillary acidic protein (GFAP) and doublecortin (DCX) markers, respectively. The RMS of aged mice preserved GFAP+ cells that were stained more intensively than those from young mice (Figures 1C,D). In contrast, the expression of DCX was severely reduced during aging (Figures 1E,F). This data indicates that the aged brain maintains the astrocytes that constitute gliotubes in the young brain, but it does not preserve chains of migrating neuroblasts.

**Figure 2:**
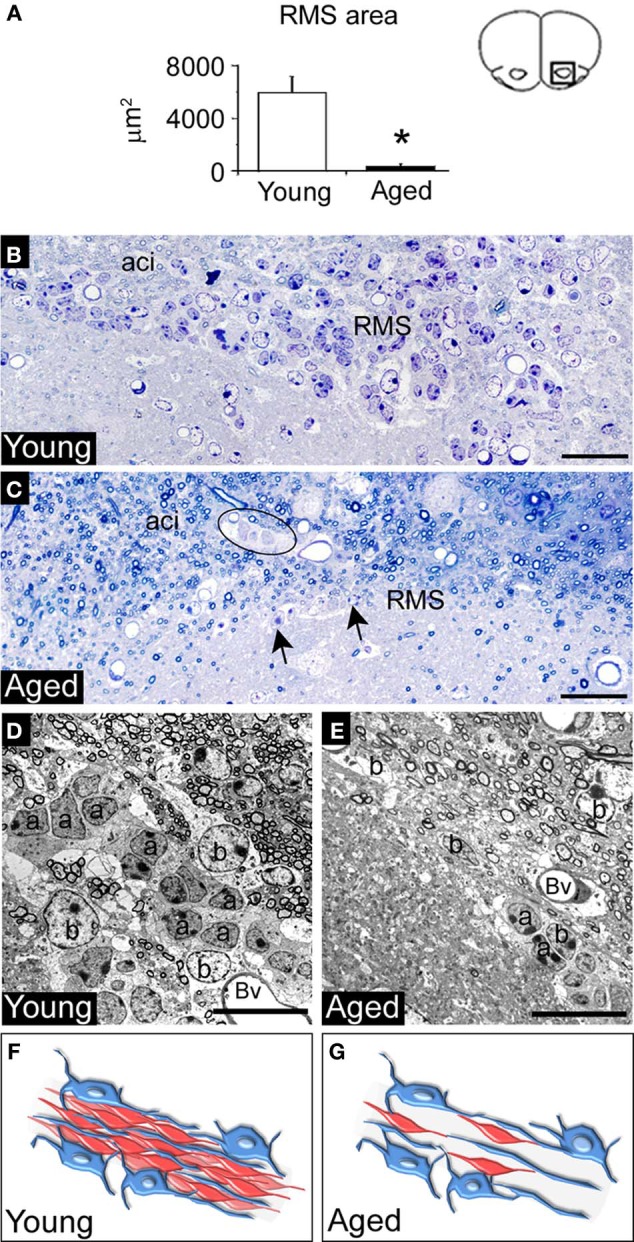
**Cytoarchitecture of aged RMS reveals a loss of migrating neuroblasts into the gliotubes.** Analysis of the RMS by using light and electron microscopy. **(A)** Bar graph depicting a significant reduction of the area occupied by the RMS in aged mice. **(B)** Semithin section of the young RMS showed multiples chains of neuroblasts. **(C)** Semithin section of the aged RMS showed a notable loss of migratory chains, but dispersed cells remained (arrows). Note the presence of groups of neuroblasts and astrocytes within the intrabulbar part of the anterior commissure (delineated). **(D)** Electron microscopy image of a young RMS showing a detail of the neuroblasts chains surrounded by astrocytic gliotubes. **(E)** Electron microscopy image showing a detail of the aged RMS, where neuroblasts were severely reduced. **(F)** Schematic representation of the RMS cytoarchitecture in young mice. Chains of neuroblasts (red) migrating through gliotubes, which were formed by astrocytes (blue). **(G)** Schematic representation of the RMS cytoarchitecture in aged mice. Note the loss of neuroblast migrating through the gliotubes. a, neuroblast; aci, intrabulbar part of the anterior commissure; b, astrocyte; Bv, blood vessel. Scale bar: **B,C** = 20 μm, **D,E** = 10 μm. ^*^p < 0.01.

To investigate these findings further we used transmission electron microscopy. The analysis of RMS coronal sections revealed a notable loss of cells during aging, which resulted in a significant decrease of the area occupied by the RMS compared to young animals (Young 1217.7 μm^2^ vs. Aged 218.7 μm^2^, *p* = 0.003) (Figures 2A–C). Remaining cells in the aged RMS were found to form small groups of cells that appeared isolated. Unlike the young mice, occasional cells were found in the intrabulbar part of the anterior commissure of the aged brain, where axons are located (Figure 2C). At higher magnifications, we detected that the reduction in the area occupied by the RMS was primarily due to a loss of migrating neuroblasts (Figures 2D–G). We did not observe ultrastructural differences in the remaining neuroblasts of the aged RMS compared to those from young mice. However, we found abundant dense bodies in the cytosol of astrocytes and frequent microglial cells close to the RMS in the aged brain (Figure S3).

### Proliferative cells within the RMS decrease in the aged brain

**Figure 3:**
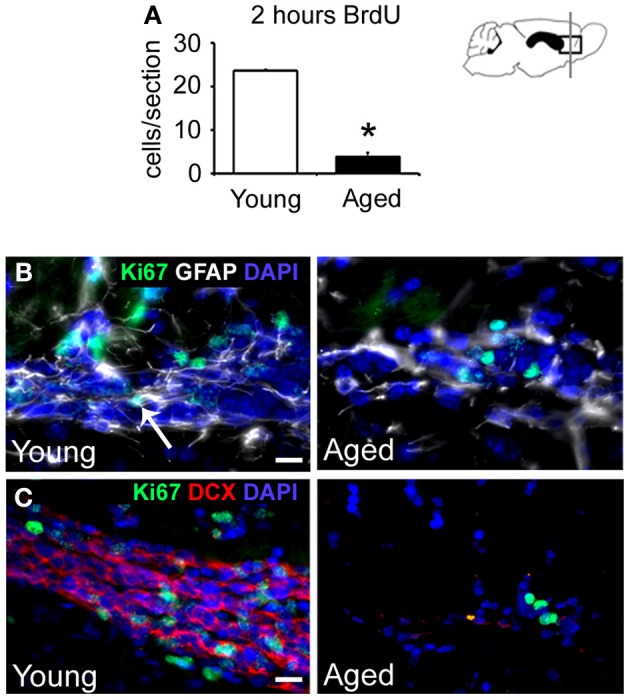
**Aging decreases the population of proliferating cells in the RMS. (A)** Bar graph depicting the number of BrdU+ cells in coronal sections of the RMS, 2 h after BrdU administration. Note the significant decrease of proliferative cells in the aged RMS. **(B)** Immunoassay against Ki67 (green) and GFAP (white) in sagittal sections of the RMS. The number of Ki67+ was drastically reduced in aged mice, while GFAP+ cells were maintained during aging. Note the presence of double Ki67/GFAP+ cells (arrow) in the young RMS. **(C)** Immunoassay against Ki67 (green) and DCX (red) in sagittal sections of the RMS. The number of both Ki67+ and DCX+ cells was drastically reduced in aged mice. Scale bar: **B,C** = 10 μm. ^*^p < 0.01.

**Figure 4:**
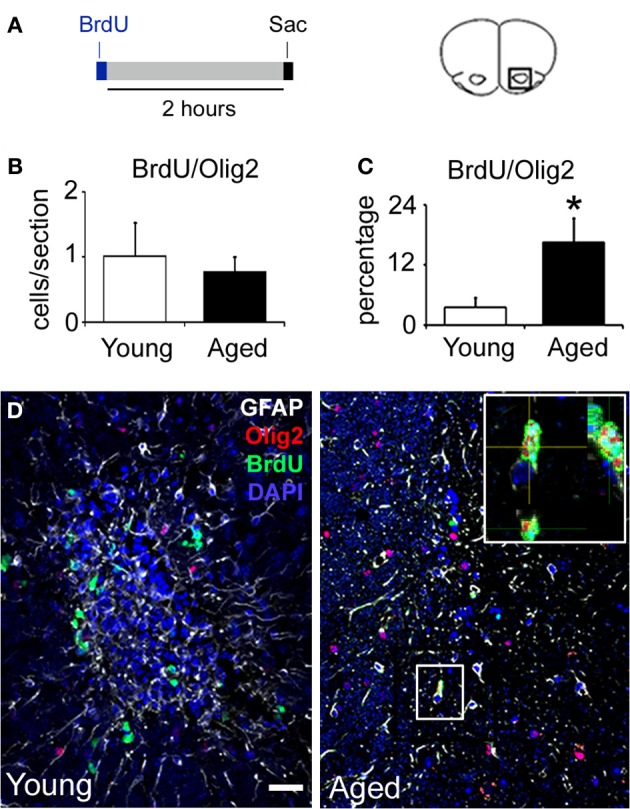
**A high percentage of the RMS proliferative cells pertain to the oligodendroglial lineage. (A)** Animals received a single dose of BrdU and were euthanized 2 h after. **(B)** Bar graph depicting the number of BrdU/Olig2+ cells in the RMS. Note that there is not a significant difference between young and aged mice. **(C)** Bar graph depicting the percentage of BrdU/Olig2+ cells in the RMS. **(D)** Immunostaining against BrdU (green), Olig2 (red), and GFAP (white) in coronal sections of the RMS. Note the presence of cells co-expressing BrdU and Olig2 markers. Sac, sacrifice. Scale bar: **C** = 20 μm. ^*^p < 0.05.

To study the proliferative capacity of remaining cells in the aged RMS, animals received a single dose of 5-bromo-2-deoxyuridine (BrdU) 2 h before sacrifice. We observed an 83% reduction in the number of BrdU+ cells per section in the RMS of aged mice (Young 23.6 ± 0.4 cells vs. Aged 4 ± 0.8 cells, *p* < 0.001) (Figure 3A). These proliferative cells were found in small groups of cells that were preserved in the aged RMS. Given that BrdU is only incorporated by cells in S-phase, we also used the proliferation marker Ki67 that is present during all active phases of the cell cycle (G1, S, G2, and mitosis). Consistently, we observed frequent Ki67+ cells in the young RMS, while they were occasional in aged mice (*n* = 3 in all groups), supporting the results from the BrdU assay (Figures 3B,C). To determine the identity of these proliferative cells, we performed double immunostaining against Ki67-GFAP or Ki67-DCX. In the aged RMS, some proliferative cells were found to express GFAP (Figure 3B and Figure S4), however, proliferating DCX+ cells were not detected (Figure 3C). In addition, to evaluate if proliferative cells were from the oligodendroglial lineage, we used the transcription factor Olig2. Surprisingly, we found that both groups of animals presented an equal number of BrdU/Olig2+ cells per section (Young 1.01 ± 0.5 cells vs. Aged 0.8 ± 0.2 cells, *p* = 0.692). Given that the overall number of BrdU+ cells declines over time, there was a resulting increase in the proportion of BrdU+ cells that expressed the Olig2 marker in the aged RMS (Young 3.5 ± 1.9% vs. Aged 16.5 ± 4.7%, *p* = 0.0117) (Figures 4A–D). These findings suggest that remaining proliferative cells in the aged RMS could be supporting oligodendrogenesis.

### Newly generated cells in the aged RMS become oligodendrocytes

**Figure 5:**
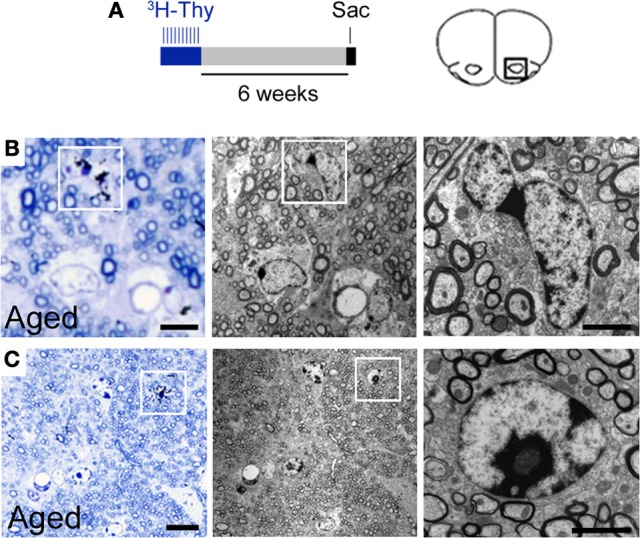
**Thymidine incorporation assay supports the generation of new oligodendrocytes in the aged RMS. (A)** Animals received ten consecutive doses of 3H-Thy cells (dose/day) and were euthanized 6 weeks after. **(B)** Light and electron microscopy images showing a labeled cell (boxes) with irregular contours and light cytoplasm, with few intermediate filaments. The nucleus was fusiform and contained dense chromatin that was peripherally distributed. This cell contained features which bore a resemblance to astrocytes and oligodendrocytes. **(C)** Light and electron microscopy images showing a typical oligodendrocyte labeled with 3H-Thy (boxes). Sac, sacrifice. Scale bar: **B** = 5 μm, detail in **(B)** 2 μm, **C** = 10 μm, detail in **(C)** = 2 μm.

In order to evaluate the proliferative potential of the RMS cells in a longer period of time and to determine the fate of the newly generated cells by ultrastructural analysis, a group of mice was injected with tritiated thymidine (^3^H-Thy, 1 dose/day) over a 10-day period and euthanized after 6 weeks (Figure 5A). The ^3^H-Thy+ cells found in the aged RMS displayed irregular contours and light cytoplasm with few intermediate filaments. Their nuclei were fusiform and contained dense, peripherally-distributed chromatin. These ^3^H-Thy+ cells had ultrastructural features bearing a resemblance to astrocytes and oligodendrocytes (Figure 5B). We also detected ^3^H-Thy+ oligodendrocytes displaying a smooth contour, round nucleus, and dark cytoplasm that contained short cisternae of rough endoplasmic reticulum and an absence of intermediate filaments (Figure 5C). The lack of dense bodies in the cytoplasm of these cells discarded the possibility of them being microglia cells. Neuroblasts labeled with ^3^H-Thy were not detected in any group, likely due to the fact that these cells had already migrated to the OB at the time of euthanasia. Together, these findings support the hypothesis that the incorporation of neuroblasts into the OB is impaired during aging, while oligodendrogenesis is preserved.

### Newly generated cells in the aged OB support the oligodendroglial lineage

**Figure 6:**
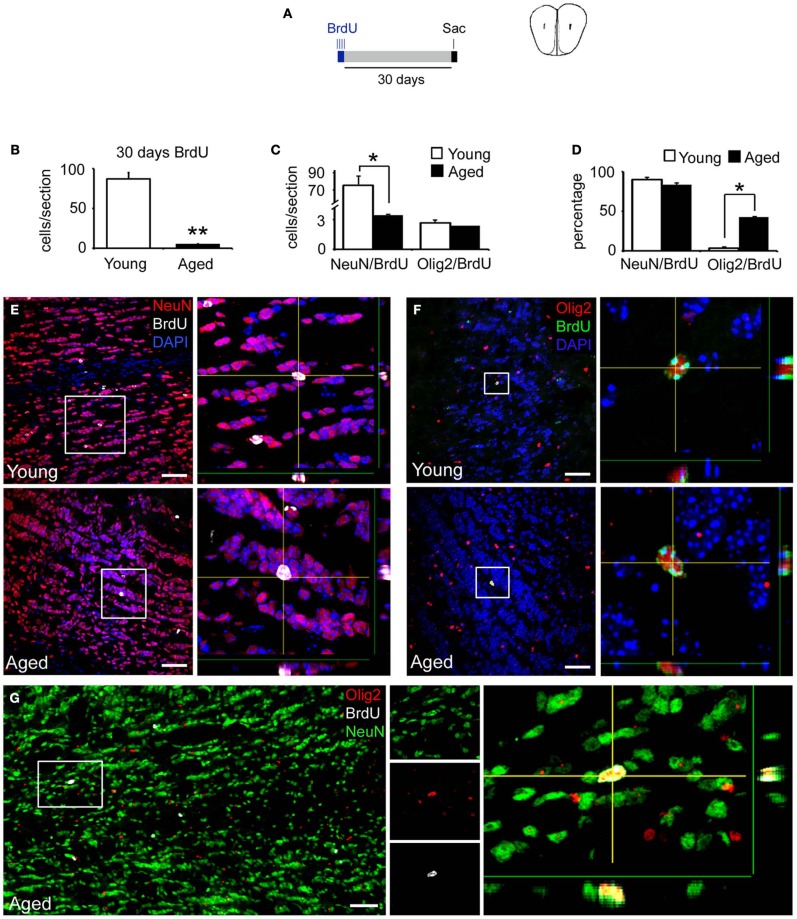
**Aging decreases neurogenesis, but not oligodendrogenesis in the OB. (A)** The animals received 4 doses of BrdU, separated by 2 h, and were euthanized 30 days after. **(B)** Bar graph depicting a significant reduction of BrdU+ cells in the aged OB, 30 days after treatment. **(C)** Bar graph depicting the number of NeuN and Olig2+ cells co-expressing BrdU marker. Note the decrease of newly generated neurons in aged mice, while the number of new oligodendrocytes remained unchanged. **(D)** Bar graph depicting the percentage of NeuN and Olig2+ cells co-expressing BrdU marker. **(E)** Immunostaining against NeuN (red) and BrdU (white) markers representing bar graphs in **(B,C)**. **(F)** Immunostaining against Olig2 (red) and BrdU (green) markers representing bar graph in **(B,C)**. **(G)** Immunostaining against BrdU (white), Olig2 (red), and NeuN (green) showing BrdU/Olig2/NeuN+ cells in the OB of aged mice. Sac, sacrifice. Scale bar: 40 μm. ^*^p < 0.05, ^**^p < 0.01.

To evaluate if neurogenesis in the OB was disrupted in the aged brain, animals were injected with 4 doses of BrdU, each dose injected with a 2 h interval, and euthanized after 30 days (Figure 6A). Quantitative analysis of OB coronal sections showed a significant decrease of BrdU+ cells in the aged group (Young 87.31 ± 7.84 cells vs. Aged 5.31 ± 0.33 cells, *p* < 0.001; Figure 6B). In accordance, a decrease in the neuroblast population was observed in the aged OB, which was more pronounced in the medial region (Figure S5). To verify if newly generated cells in the OB were differentiating into mature neurons, we performed a double immunostaining against BrdU and NeuN markers. The number of BrdU/NeuN+ cells per section was significantly reduced in aged mice (Young 75.6 ± 13.01 cells vs. Aged 3.5 ± 0.3 cells, *p* = 0.01), while the percentage of BrdU+ cells that expressed NeuN did not differ across the two experimental groups (Young 90.12 ± 2.5% vs. Aged 83.7 ± 2.9%, *p* = 0.228). Next, the production of new cells from the oligodendroglial lineage was evaluated using the Olig2 marker. Contrarily to the decrease in neurogenesis, aged mice displayed an increase in the percentage of cells that co-expressed BrdU and Olig2 markers (Young 3.5 ± 1.1% vs. Aged 42.7 ± 0.7%, *p* < 0.001), but did not show a difference in the number of BrdU/Olig2+ cells (Young 2.7 ± 0.6 cells vs. Aged 2.4 ± 0.2 cells, *p* = 0.675) (Figures 6C–F). The generation of new oligodendroglial cells was also examined in the corpus callosum, striatum, and SVZ of the aged mice, finding a consistent increase in the proportion of cells co-expressing BrdU and Olig2 in the SVZ (Figure S6). Interestingly, the aged OB showed occasional cells co-expressing BrdU, Olig2, and NeuN markers (Figure 6G). Despite the reduction in neurogenesis with aging, these results suggest that the production of oligodendrocytes is maintained across the aged SVZ-OB system.

In order to test the possibility that the new cells found in the OB were endogenously generated from local progenitors (Gritti et al., 2002), we examined the OB of animals that received a single dose of BrdU and were euthanized 2 h later. Immunostaining against BrdU revealed that proliferation was significantly decreased in the aged OB (Young 21.1 ± 4.6 cells vs. Aged 5.1 ± 0.4 cells, *p* < 0.001). This reduction of proliferative cells was more pronounced in the medial region of the OB, where migrating neuroblasts arrive. Interestingly, proliferative cells were found to express Olig2 in the young OB, but not in the aged OB (Figure S7). These results suggest that the new oligodendroglial cells within the aged OB were not originated from local progenitors. Nonetheless, more experiments would be required to conclusively study the origin of the new oligodendroglial cells.

## Discussion

During aging, the SVZ undergoes a severe decline in its proliferative capacity which subsequently affects neurogenesis in the OB (Maslov et al., 2004; Luo et al., 2006; Bouab et al., 2011; McGinn et al., 2012). However, the age-related changes induced in the migratory pathway that the newly generated cells follow had not been completely described. In this study we present important findings on the effects of aging across the SVZ-OB system. We demonstrated that proliferation declines at the RMS and OB levels during aging, similar to the SVZ. We observed a decrease in the number of neuroblasts migrating across the RMS and an altered cellular organization of this migratory pathway. Finally, while we confirmed an OB neurogenesis decline with aging, oligodendrogenesis across the RMS was surprisingly not compromised.

Newly generated cells in the SVZ migrate a long distance through the RMS before reaching the OB, where they differentiate into interneurons to replace old cells (Lois and Alvarez-Buylla, 1994). The SVZ maintains this neurogenic capacity throughout life, but the number of cells that ultimately integrate into the OB decrease over time (Bouab et al., 2011; Shook et al., 2012). Consistent with these findings, we described a remarkable depletion of the migratory chains of neuroblasts in the SVZ during senescence, which was mimicked in the RMS. While the gliotubes of astrocytic cells were maintained in the aged brain, the number of migrating neuroblasts was drastically reduced. Subsequently, we found a decrease of newly generated cells in the aged OB, in accordance with previous publications (Bouab et al., 2011; Shook et al., 2012). These effects on OB neurogenesis could be a direct consequence of altering the SVZ niche. Prior studies from our group support this hypothesis as we previously demonstrated that the disruption of the SVZ by exposure to N-ethyl-N-nitrosourea leads to an impairment in the incorporation of new cells into the OB (Capilla-Gonzalez et al., 2012). Similarly, the radiation impact on the SVZ neurogenic niche interrupted neuroblast migration to the OB (Lazarini et al., 2009; Achanta et al., 2012). Another explanation for OB neurogenesis decline is that aging could impair the migratory capacity of neuroblasts, however, a recent publication suggests that the capacity of neuroblasts to exit the SVZ and reach the OB is not affected by age (Mobley et al., 2013). Based on these reports, we consider that SVZ-derived cells are able to migrate in the aged brain, but the number of cells that finally incorporate into the OB is drastically reduced due to the age-related effects on the SVZ niche.

Contrary to the decline in neurogenesis during senescence, we demonstrated, for the first time, that the production of oligodendroglial cells is maintained in the aged SVZ-OB system. An increase in oligodendrocyte-generation has been observed in other regions of the central nervous system, such as the spinal cord and neocortex of rodents (Levison et al., 1999; Lasiene et al., 2009), or the fornix of monkeys (Peters et al., 2010). Concurrent with these reports, a more recent study indicate that NSCs, derived from young and middle-aged SVZ, present similar ability to produce oligodendrocytes *in vitro,* when they were differentiated in absence of exogenous growth factors (Bouab et al., 2011). In our study, we found that the capacity of the SVZ to produce Olig2+ cells *in vivo* is not diminished by aging. The maintained SVZ ability to produce Olig2+ cells in the aged brain could subsequently help to preserve the oligodendrogenesis in the RMS-OB. This would require that a group of SVZ-derived cells become oligodendrocytes instead of differentiating into neurons. This idea is supported by previous reports showing that DCX+ cells from the SVZ can be redirected from neuronal to glial fates following a demyelination process, and generate new oligodendrocytes in the corpus callosum (Jablonska et al., 2010). Another possible explanation for the maintenance of the oligodendrogenesis in the aged brain is that local stem cells within the OB are contributing to the production of oligodendroglial cells (Gritti et al., 2002; Rivers et al., 2008). In fact, stem cells isolated from the OB were found to produce more oligodendrocytes *in vitro* than those from the SVZ (Gritti et al., 2002). On the other hand, it could be possible that those local precursors within the OB may support neurogenesis to serve as a compensatory mechanism for the loss of newly arriving neuroblasts from the SVZ. Indeed, it has been described that oligodendrocyte progenitors in the OB are also able to produce new neurons in transgenic mice, after the blockage of the Platelet-Derived Growth Factor Receptor-alpha (Rivers et al., 2008). In accordance with this finding, we observed cells co-expressing BrdU, NeuN, and Olig2 markers in the aged OB. However, the reduced number of proliferative cells detected in the OB of aged mice conflicts with the possibility that most newly generated cells, either neurons or oligodendrocytes, were produced from local precursors instead of being SVZ-derived. These modifications in cell production may respond to age-related changes in cell signaling pathways. For instance, the balance between neurogenesis and gliogenesis in the SVZ niche is controlled by the BMP and noggin signaling (Lim et al., 2000; Mekki-Dauriac et al., 2002; Bilican et al., 2008). SVZ astrocytes express BMP inducing gliogenesis, while ependymal cells express noggin that promotes neurogenesis. Since the SVZ niche is disrupted during aging (Bouab et al., 2011; Shook et al., 2012), the BMP-noggin balance could be modified and contribute to the neurogenesis decline and favor oligodendrogenesis. Further studies are required to fully understand the mechanisms associated to the age-related changes described here.

Although the existence of the RMS in the adult human brain remains highly controversial (Sanai et al., 2004, 2007; Curtis et al., 2007; Kam et al., 2009; Wang et al., 2011), the presence of neural stem cells within the SVZ is widely accepted (Sanai et al., 2004; Quinones-Hinojosa et al., 2006). Nevertheless, the function of the newly generated cells in the human brain is currently unknown. A recent publication demonstrated that the postnatal production of new neurons in humans is minimal, while there is a continuous turnover of non-neuronal cells, which could contribute to the production of oligodendrocytes (Bergmann et al., 2012). Hence, the neurogenic system of aged mice becomes a better model for comparisons with adult human beings than the neurogenic system from young rodents. The maintained oligodendrogenesis provides new insight into the relevance of the oligodendrocytes throughout life, which could be a response to myelin damaged during aging (Nait-Oumesmar et al., 1999; Jablonska et al., 2010; Gonzalez-Perez and Alvarez-Buylla, 2011; Capilla-Gonzalez et al., in press). Moreover, oligodendrogenesis could be beneficial for the proper function of the remaining neurons in the aged brain. All of these possible functions could be crucial to the survival and maturation of newly generated oligodendrocytes.

In conclusion, we have demonstrated that the decrease of SVZ proliferation during aging is mimicked in the RMS-OB system. Neuroblast migratory chains are severely disrupted across the whole RMS of aged mice, consistent with OB neurogenesis impairment. Furthermore, oligodendrogenesis is maintained in the SVZ-OB system of the aged brain, but the origin of these new cells is still unknown. These findings provide new insight into the events occurring during aging on the genesis of new cells. Modifying the source and subsequent fate of these newly generated cells in the aged brain could serve as the foundation for new therapeutic strategies for brain disorders associated with senescence.

## Experimental procedures

### Animals

The animals used were 2 and 24-month-old C57BL/6 male mice (*n*_young_ = 20, *n*_aged_ = 22) from National Institute on Aging (Baltimore, MD, USA) and Charles River Laboratory (Barcelona, Spain). Animals were housed under a 12-h light/dark cycle with food and water available *ad libitum*. All animal procedures were performed according to the European Communities Council (86/609/EEC) guidelines and approved by the Johns Hopkins Animal Care and Use Committee and followed standard animal care and use protocols.

### Administration of 5-bromo-2-deoxyuridine

5-bromo-2-deoxyuridine (BrdU, Sigma Aldrich, Dorset, UK) is an exogenous marker, which is incorporated into the newly synthesized DNA of replicating cells, during the S phase. To study the proliferation, animals received a single intraperitoneal injection of BrdU (50 mg/Kg b.wt.) and were euthanized 2 h after (*n*_young_ = 4, *n*_aged_ = 6). To assess cell migration, animals received 4 intraperitoneal injections of BrdU, separated by 2 h, and were euthanized 30 days later (*n*_young_ = 3, *n*_aged_ = 3).

### Tritiated thymidine administration

For ultrastructural identification of the proliferative cells and newly generated cells, animals received a single daily dose of 1.67 μl/g b.wt. of 1 mCi tritiated thymidine (^3^H-Thy; specific activity 5 Ci/mmol; Amersham Biosciences, Uppsala, Sweden), for 10 consecutive days, and were euthanized 6 weeks after the last injection (*n*_young_ = 4, *n*_aged_ = 4).

### Brain tissue fixation

Animals were anesthetized by an intraperitoneal injection of 2:1 ketamine/xylazine (5 μl/g of weight) and subjected to an intracardiac perfusion using a peristaltic pump. As fixative, we used 4% paraformaldehyde for immunohistochemistry, or 2% paraformaldehyde and 2.5% glutaraldehyde for electron microscopy. Prior brain dissection, heads were removed and post-fixed in the same fixative overnight.

### Immunohistochemistry

After post-fixation, brains were washed in 0.1 M PB and cut into serial 10 μm thick coronal (*n*_young_ = 7, *n*_aged_ = 9) or sagittal (*n*_young_ = 3, *n*_aged_ = 3) sections using a cryostat (Leica, CM 1900). One series (5–8 sections) from each animal was used in each immunostaining. Sections were incubated in blocking solution for 1 h at room temperature, followed by overnight incubation at 4°C with primary antibodies (see Table S1). Then, sections were washed and incubated with the appropriate secondary antibodies conjugated with either biotin or fluorophores. After the secondary biotinylated antibody, sections were incubated with ABC Elite complex (Vector, Burlingame, CA, USA) and treated with diaminobenzidine (DAB, 0.05%; Sigma Aldrich). Measurement of BrdU incorporation during DNA synthesis was carried out in coronal sections by quantification of BrdU+ cells under an Eclipse E200 light microscope (Nikon, Tokyo, Japan), and were expressed as cells/section. Fluorescence samples were examined under an Olympus IX81 confocal microscope and imaged using the Olympus Fluoview software version 3.1 (Center Valley, PA, USA). Three-dimensional images were obtained using the Slidebook™ software (Intelligent Imaging Innovations, Denver, CO, USA). Quantification of BrdU double immunostaining with NeuN or Olig2 was expressed as double+ cells per section and as percentage (100 × BrdU-NeuN+ cells/total BrdU+ cells and 100 × BrdU-Olig2+ cells/total BrdU+ cells).

### Transmission electron microscopy

Post-fixated brains were washed in 0.1 M phosphate buffer (PB; pH 7.4), cut into 200 μm sections with a VT 1000 M vibratome (Leica, Wetzlar, Germany) and treated with 2% osmium tetraoxide in 0.1 M PB for 2 h. Then sections were rinsed, dehydrated through increasing ethanol solutions and stained in 2% uranyl acetate at 70% ethanol. Following dehydration, slices were embedded in araldite (Durcupan, Fluka BioChemika, Ronkokoma, NY, USA). To study cell organization of the RMS, we cut serial 1.5 μm semithin sections with a diamond knife and stained them with 1% toluidine blue. To identify individual cell types, 60–70 nm ultrathin sections were cut with a diamond knife, stained with lead citrate, and examined under a Spirit transmission electron microscope (FEI Tecnai, Hillsboro, OR, USA). Changes in the area of the RMS were determined by measuring the area occupied by the RMS in semithin sections of 3 different levels per animal (*n*_young_ = 6, *n*_aged_ = 6), using light microscopy. The analysis was performed with Image Tool software (Evans Technology, Roswell, GA, USA).

### Tritiated thymidine autoradiography

Brains treated with ^3^H-Thy were processed for transmission electron microscopy as described above. Subsequently, semithin sections were dipped in LM-1 hypercoat emulsion (Amersham Biosciences), dried in the dark, and stored at 4°C for a month (Doetsch et al., 1997). Autoradiography was developed using standard methods and counterstained with 1% toluidine blue. Selected semithin sections, with a total of 40 labeled cells, were processed for ultrathin sections to be analyzed using electron microscopy.

### Statistical analysis

Data were expressed as mean ± SEM. After testing for normal distribution with Shapiro–Wilke Test, a Student's *t*-test was performed using SigmaPlot 11.0 software (Jandel Scientific, San Rafael, CA, USA). For samples that were not normally distributed, the non-parametric Mann Whitney *U*-Test was used. Differences were considered significant at a *p*-value lower than 0.05.

## Author contributions

Vivian Capilla-Gonzalez, collection and assembly of data, concept and design, data analysis and interpretation, manuscript writing, final approval of manuscript; Arantxa Cebrian-Silla and Hugo Guerrero-Cazares, collection of data, data analysis and interpretation, final approval of manuscript; Jose M. Garcia-Verdugo and Alfredo Quiñones-Hinojosa, concept and design, data analysis and interpretation, final approval of manuscript, financial support.

## Conflict of interest statement

The authors declare that the research was conducted in the absence of any commercial or financial relationships that could be construed as a potential conflict of interest.

## References

[B1] Achanta P. Capilla-Gonzalez V. Purger D. Reyes J. Sailor K. Song H. (2012). Subventricular zone localized irradiation affects the generation of proliferating neural precursor cells and the migration of neuroblasts. Stem Cells 30, 2548–2560 10.1002/stem.1214 22948813 PMC3991482

[B2] Alvarez-Buylla A. Garcia-Verdugo J. M. (2002). Neurogenesis in adult subventricular zone. J. Neurosci. 22, 629–634 11826091 10.1523/JNEUROSCI.22-03-00629.2002 PMC6758521

[B3] Bergmann O. Liebl J. Bernard S. Alkass K. Yeung M. S. Steier P. (2012). The age of olfactory bulb neurons in humans. Neuron 74, 634–639 10.1016/j.neuron.2012.03.030 22632721

[B4] Bilican B. Fiore-Heriche C. Compston A. Allen N. D. Chandran S. (2008). Induction of Olig2 precursors by FGF involves BMP signalling blockade at the Smad level. PLoS ONE 3:e2863 10.1371/journal.pone.0002863 18682850 PMC2483937

[B5] Bouab M. Paliouras G. N. Aumont A. Forest-Berard K. Fernandes K. J. (2011). Aging of the subventricular zone neural stem cell niche: evidence for quiescence-associated changes between early and mid-adulthood. Neuroscience 173, 135–149 10.1016/j.neuroscience.2010.11.032 21094223

[B6] Capilla-Gonzalez V. Gil-Perotin S. Ferragud A. Bonet-Ponce L. Canales J. J. Garcia-Verdugo J. M. (2012). Exposure to N-ethyl-N-nitrosourea in adult mice alters structural and functional integrity of neurogenic sites. PLoS ONE 7:e29891 10.1371/journal.pone.0029891 22238669 PMC3251592

[B7] Capilla-Gonzalez V. Guerrero-Cazares H. Bonsu J. M. Gonzalez-Perez O. Achanta P. Wong J. (in press). The subventricular zone is able to respond to a demyelinating lesion after localized radiation. Stem Cells. 10.1002/stem.1519 PMC4879590 24038623

[B8] Carleton A. Petreanu L. T. Lansford R. Alvarez-Buylla A. Lledo P. M. (2003). Becoming a new neuron in the adult olfactory bulb. Nat. Neurosci. 6, 507–518 10.1038/nn1048 12704391

[B9] Conover J. C. Shook B. A. (2011). Aging of the subventricular zone neural stem cell niche. Aging Dis. 2, 49–63 22396866 PMC3295044

[B10] Curtis M. A. Kam M. Nannmark U. Anderson M. F. Axell M. Z. Wikkelso C. (2007). Human neuroblasts migrate to the olfactory bulb via a lateral ventricular extension. Science 315, 1243–1249 10.1126/science.1136281 17303719

[B11] Doetsch F. Garcia-Verdugo J. M. Alvarez-Buylla A. (1997). Cellular composition and three-dimensional organization of the subventricular germinal zone in the adult mammalian brain. J. Neurosci. 17, 5046–5061 9185542 10.1523/JNEUROSCI.17-13-05046.1997 PMC6573289

[B12] Gonzalez-Perez O. Alvarez-Buylla A. (2011). Oligodendrogenesis in the subventricular zone and the role of epidermal growth factor. Brain Res. Rev. 67, 147–156 10.1016/j.brainresrev.2011.01.001 21236296 PMC3109119

[B13] Gonzalez-Perez O. Romero-Rodriguez R. Soriano-Navarro M. Garcia-Verdugo J. M. Alvarez-Buylla A. (2009). Epidermal growth factor induces the progeny of subventricular zone type B cells to migrate and differentiate into oligodendrocytes. Stem Cells 27, 2032–2043 10.1002/stem.119 19544429 PMC3346259

[B14] Gritti A. Bonfanti L. Doetsch F. Caille I. Alvarez-Buylla A. Lim D. A. (2002). Multipotent neural stem cells reside into the rostral extension and olfactory bulb of adult rodents. J. Neurosci. 22, 437–445 11784788 10.1523/JNEUROSCI.22-02-00437.2002 PMC6758684

[B15] Guerrero-Cazares H. Gonzalez-Perez O. Soriano-Navarro M. Zamora-Berridi G. Garcia-Verdugo J. M. Quinones-Hinojosa A. (2011). Cytoarchitecture of the lateral ganglionic eminence and rostral extension of the lateral ventricle in the human fetal brain. J. Comp. Neurol. 519, 1165–1180 10.1002/cne.22566 21344407 PMC3886186

[B16] Imayoshi I. Sakamoto M. Ohtsuka T. Takao K. Miyakawa T. Yamaguchi M. (2008). Roles of continuous neurogenesis in the structural and functional integrity of the adult forebrain. Nat. Neurosci. 11, 1153–1161 10.1038/nn.2185 18758458

[B17] Jablonska B. Aguirre A. Raymond M. Szabo G. Kitabatake Y. Sailor K. A. (2010). Chordin-induced lineage plasticity of adult SVZ neuroblasts after demyelination. Nat. Neurosci. 13, 541–550 10.1038/nn.2536 20418875 PMC4059417

[B18] Kam M. Curtis M. A. McGlashan S. R. Connor B. Nannmark U. Faull R. L. (2009). The cellular composition and morphological organization of the rostral migratory stream in the adult human brain. J. Chem. Neuroanat. 37, 196–205 10.1016/j.jchemneu.2008.12.009 19159677

[B19] Kelsch W. Sim S. Lois C. (2010). Watching synaptogenesis in the adult brain. Annu. Rev. Neurosci. 33, 131–149 10.1146/annurev-neuro-060909-153252 20572770

[B20] Lasiene J. Matsui A. Sawa Y. Wong F. Horner P. J. (2009). Age-related myelin dynamics revealed by increased oligodendrogenesis and short internodes. Aging Cell 8, 201–213 10.1111/j.1474-9726.2009.00462.x 19338498 PMC2703583

[B21] Lazarini F. Lledo P. M. (2011). Is adult neurogenesis essential for olfaction? Trends Neurosci. 34, 20–30 10.1016/j.tins.2010.09.006 20980064

[B22] Lazarini F. Mouthon M. A. Gheusi G. De Chaumont F. Olivo-Marin J. C. Lamarque S. (2009). Cellular and behavioral effects of cranial irradiation of the subventricular zone in adult mice. PLoS ONE 4:e7017 10.1371/journal.pone.0007017 19753118 PMC2737283

[B23] Levison S. W. Young G. M. Goldman J. E. (1999). Cycling cells in the adult rat neocortex preferentially generate oligodendroglia. J. Neurosci. Res. 57, 435–446 10.1002/(SICI)1097-4547(19990815)57:4<435::AID-JNR3>3.0.CO;2-L 10440893

[B24] Lim D. A. Tramontin A. D. Trevejo J. M. Herrera D. G. Garcia-Verdugo J. M. Alvarez-Buylla A. (2000). Noggin antagonizes BMP signaling to create a niche for adult neurogenesis. Neuron 28, 713–726 10.1016/S0896-6273(00)00148-3 11163261

[B25] Lois C. Alvarez-Buylla A. (1994). Long-distance neuronal migration in the adult mammalian brain. Science 264, 1145–1148 10.1126/science.8178174 8178174

[B26] Lois C. Garcia-Verdugo J. M. Alvarez-Buylla A. (1996). Chain migration of neuronal precursors. Science 271, 978–981 10.1126/science.271.5251.978 8584933

[B27] Luo J. Daniels S. B. Lennington J. B. Notti R. Q. Conover J. C. (2006). The aging neurogenic subventricular zone. Aging Cell 5, 139–152 10.1111/j.1474-9726.2006.00197.x 16626393

[B28] Luskin M. B. Zigova T. Soteres B. J. Stewart R. R. (1997). Neuronal progenitor cells derived from the anterior subventricular zone of the neonatal rat forebrain continue to proliferate *in vitro* and express a neuronal phenotype. Mol. Cell. Neurosci. 8, 351–366 10.1006/mcne.1996.0592 9073397

[B29] Maslov A. Y. Barone T. A. Plunkett R. J. Pruitt S. C. (2004). Neural stem cell detection, characterization, and age-related changes in the subventricular zone of mice. J. Neurosci. 24, 1726–1733 10.1523/JNEUROSCI.4608-03.2004 14973255 PMC6730468

[B30] McGinn M. J. Colello R. J. Sun D. (2012). Age-related proteomic changes in the subventricular zone and their association with neural stem/progenitor cell proliferation. J. Neurosci. Res. 90, 1159–1168 10.1002/jnr.23012 22344963 PMC3323769

[B31] Mekki-Dauriac S. Agius E. Kan P. Cochard P. (2002). Bone morphogenetic proteins negatively control oligodendrocyte precursor specification in the chick spinal cord. Development 129, 5117–5130 12399304 10.1242/dev.129.22.5117

[B32] Menn B. Garcia-Verdugo J. M. Yaschine C. Gonzalez-Perez O. Rowitch D. Alvarez-Buylla A. (2006). Origin of oligodendrocytes in the subventricular zone of the adult brain. J. Neurosci. 26, 7907–7918 10.1523/JNEUROSCI.1299-06.2006 16870736 PMC6674207

[B33] Mobley A. S. Bryant A. K. Richard M. B. Brann J. H. Firestein S. J. Greer C. A. (2013). Age-dependent regional changes in the rostral migratory stream. Neurobiol. Aging 34, 1873–1881 10.1016/j.neurobiolaging.2013.01.015 23419702 PMC3622839

[B34] Molofsky A. V. Slutsky S. G. Joseph N. M. He S. Pardal R. Krishnamurthy J. (2006). Increasing p16INK4a expression decreases forebrain progenitors and neurogenesis during ageing. Nature 443, 448–452 10.1038/nature05091 16957738 PMC2586960

[B35] Nait-Oumesmar B. Decker L. Lachapelle F. Avellana-Adalid V. Bachelin C. Van Evercooren A. B. (1999). Progenitor cells of the adult mouse subventricular zone proliferate, migrate and differentiate into oligodendrocytes after demyelination. Eur. J. Neurosci. 11, 4357–4366 10.1046/j.1460-9568.1999.00873.x 10594662

[B36] Peretto P. Merighi A. Fasolo A. Bonfanti L. (1997). Glial tubes in the rostral migratory stream of the adult rat. Brain Res. Bull. 42, 9–21 10.1016/S0361-9230(96)00116-5 8978930

[B37] Peters A. Sethares C. Moss M. B. (2010). How the primate fornix is affected by age. J. Comp. Neurol. 518, 3962–3980 10.1002/cne.22434 20737595 PMC2929937

[B38] Ponti G. Obernier K. Guinto C. Jose L. Bonfanti L. Alvarez-Buylla A. (2013). Cell cycle and lineage progression of neural progenitors in the ventricular-subventricular zones of adult mice. Proc. Natl. Acad. Sci. U.S.A. 110, E1045–E1054 10.1073/pnas.1219563110 23431204 PMC3600494

[B39] Poon A. Li Z. Wolfe G. W. Lu L. Williams R. W. Hayes N. L. (2010). Identification of a Chr 11 quantitative trait locus that modulates proliferation in the rostral migratory stream of the adult mouse brain. Eur. J. Neurosci. 32, 523–537 10.1111/j.1460-9568.2010.07316.x 20718853 PMC3382016

[B40] Quinones-Hinojosa A. Sanai N. Soriano-Navarro M. Gonzalez-Perez O. Mirzadeh Z. Gil-Perotin S. (2006). Cellular composition and cytoarchitecture of the adult human subventricular zone: a niche of neural stem cells. J. Comp. Neurol. 494, 415–434 10.1002/cne.20798 16320258

[B41] Rivers L. E. Young K. M. Rizzi M. Jamen F. Psachoulia K. Wade A. (2008). PDGFRA/NG2 glia generate myelinating oligodendrocytes and piriform projection neurons in adult mice. Nat. Neurosci. 11, 1392–1401 10.1038/nn.2220 18849983 PMC3842596

[B42] Sanai N. Berger M. S. Garcia-Verdugo J. M. Alvarez-Buylla A. (2007). Comment on “Human neuroblasts migrate to the olfactory bulb via a lateral ventricular extension.” Science 318, 393 10.1126/science.1145011 17947566

[B43] Sanai N. Nguyen T. Ihrie R. A. Mirzadeh Z. Tsai H. H. Wong M. (2011). Corridors of migrating neurons in the human brain and their decline during infancy. Nature 478, 382–386 10.1038/nature10487 21964341 PMC3197903

[B44] Sanai N. Tramontin A. D. Quinones-Hinojosa A. Barbaro N. M. Gupta N. Kunwar S. (2004). Unique astrocyte ribbon in adult human brain contains neural stem cells but lacks chain migration. Nature 427, 740–744 10.1038/nature02301 14973487

[B45] Shook B. A. Manz D. H. Peters J. J. Kang S. Conover J. C. (2012). Spatiotemporal changes to the subventricular zone stem cell pool through aging. J. Neurosci. 32, 6947–6956 10.1523/JNEUROSCI.5987-11.2012 22593063 PMC3359841

[B46] Smith C. M. Luskin M. B. (1998). Cell cycle length of olfactory bulb neuronal progenitors in the rostral migratory stream. Dev. Dyn. 213, 220–227 10.1002/(SICI)1097-0177(199810)213:2<220::AID-AJA7>3.0.CO;2-I 9786422

[B47] Wang C. Liu F. Liu Y. Y. Zhao C. H. You Y. Wang L. (2011). Identification and characterization of neuroblasts in the subventricular zone and rostral migratory stream of the adult human brain. Cell Res. 21, 1534–1550 10.1038/cr.2011.83 21577236 PMC3365638

