# Supplementary material for: The generation of oligodendroglial cells is preserved in the rostral migratory stream during aging
Source: Front Cell Neurosci. 2013 Sep 11;7:147. doi: 10.3389/fncel.2013.00147 (PMC3775451; doi:10.3389/fncel.2013.00147)
Supplement: Supplementary file 1 [file Presentation1.PDF]

Supplemental information

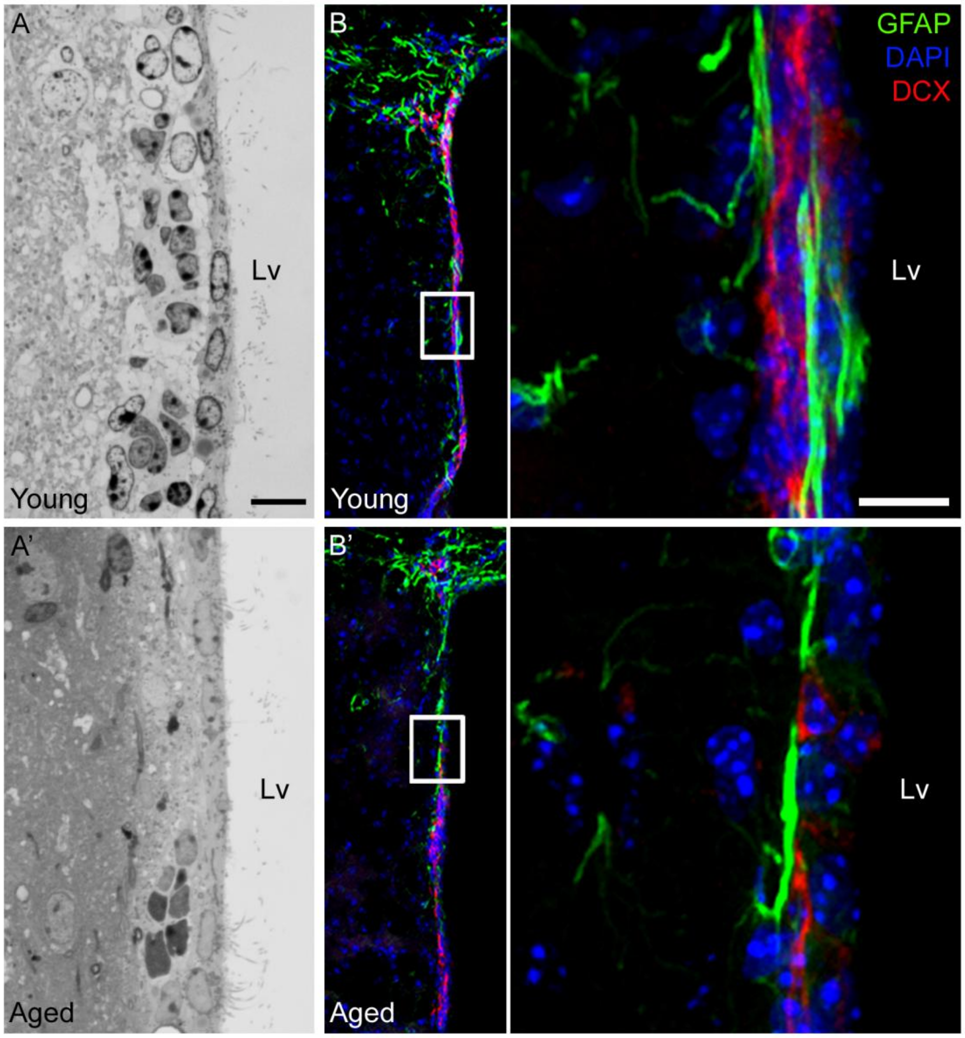

Fig. S1. Aging impairs the migratory chains of neuroblasts in the SVZ niche. (**A**) Semithin sections of the SVZ revealing a loss of migrating neuroblasts (dark cells) during aging. (**B**) Immunoassay against GFAP (green) and DCX (red) in coronal sections of the SVZ. In the young SVZ, GFAP and DCX+ cells were homogeneously distributed across the lateral ventricle wall. DCX+ cells formed chains that were surrounded by GFAP+ process, which formed the gliotubes (see detail in B). In the aged SVZ, the DCX expression was disrupted and only small chains were preserved (see detail in B'). Lv, lateral ventricle. Scale bar: A-B 10 µm.

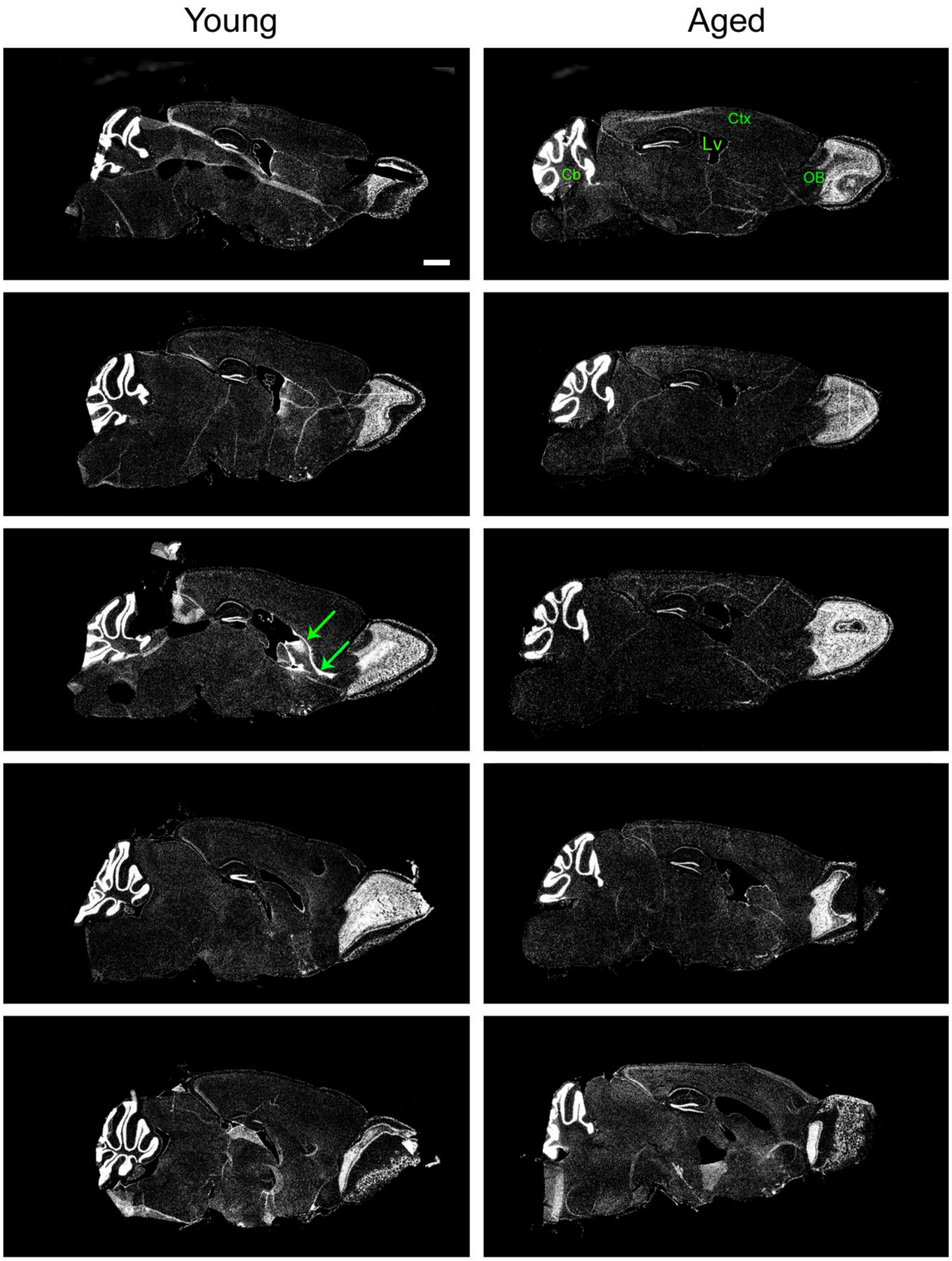

Fig. S2. The RMS tends to disappear during aging. Serial sagittal sections of the brain in young and aged mice. The DAPI staining showed a remarkable RMS (arrows) from the lateral ventricle to the OB in a particular level of the young brain. Contrarily, the RMS was not evident in any level of the aged brain. Cb, cerebellum; Ctx, cortex; Lv lateral ventricle; OB, olfactory bulb. Scale bar: 1 mm.

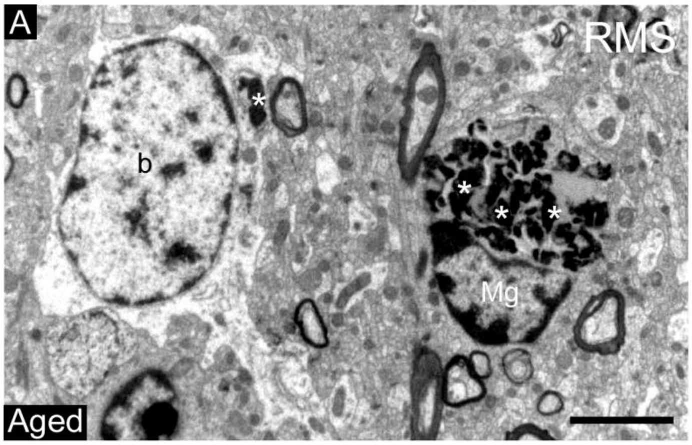

Fig. S3. The aged RMS presents astrocytes and microglia cells with dense bodies. Detail of an electron microscopy image of the aged RMS from a coronal brain section. Astrocytes and microglia cell were found with dense bodies (asterisks) in the cytoplasm. b, astrocyte; Mg, microglia cell. Scale bar: 3 µm.

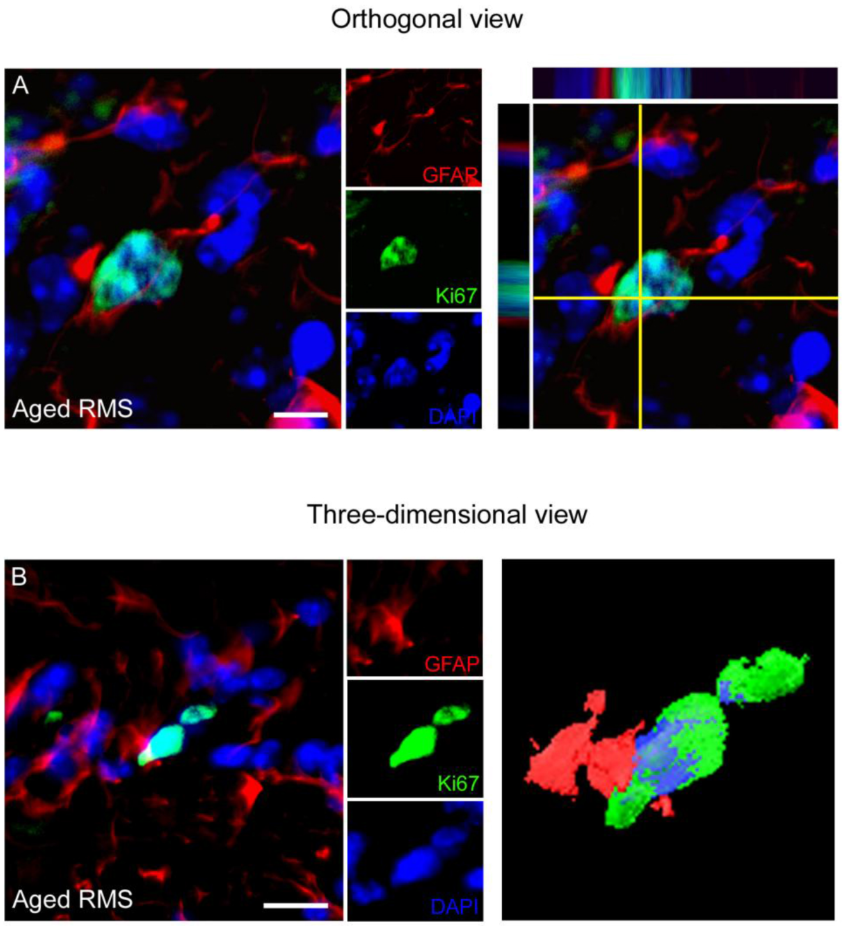

Fig. S4. Proliferating astrocytes in the RMS of the aged brain. Immunohistochemistry against GFAP (red) and Ki67 (green) markers in sagittal sections of the aged RMS. (**A**) Immunostaining images showing a cell co-expressing Ki67 and GFAP, which was examined with the orthogonal view. (**B**) Immunostaining images showing a cell co-expressing Ki67 and GFAP, which was examined with the three-dimensional view. Scale bar: A 5 µm, B 10 µm.

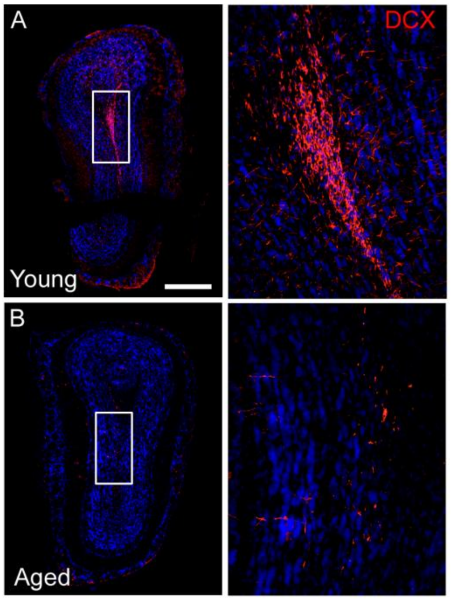

Fig. S5. The population of neuroblast within the OB decreases during aging. Immunostaining against DCX in coronal sections of the OB. (**A**) The young OB showed DCX+ cells concentrated in the medial region, from where they radially reach other OB layers. (**B**) The aged OB showed a notable decrease in the DCX expression. Scale bar: 500 µm.

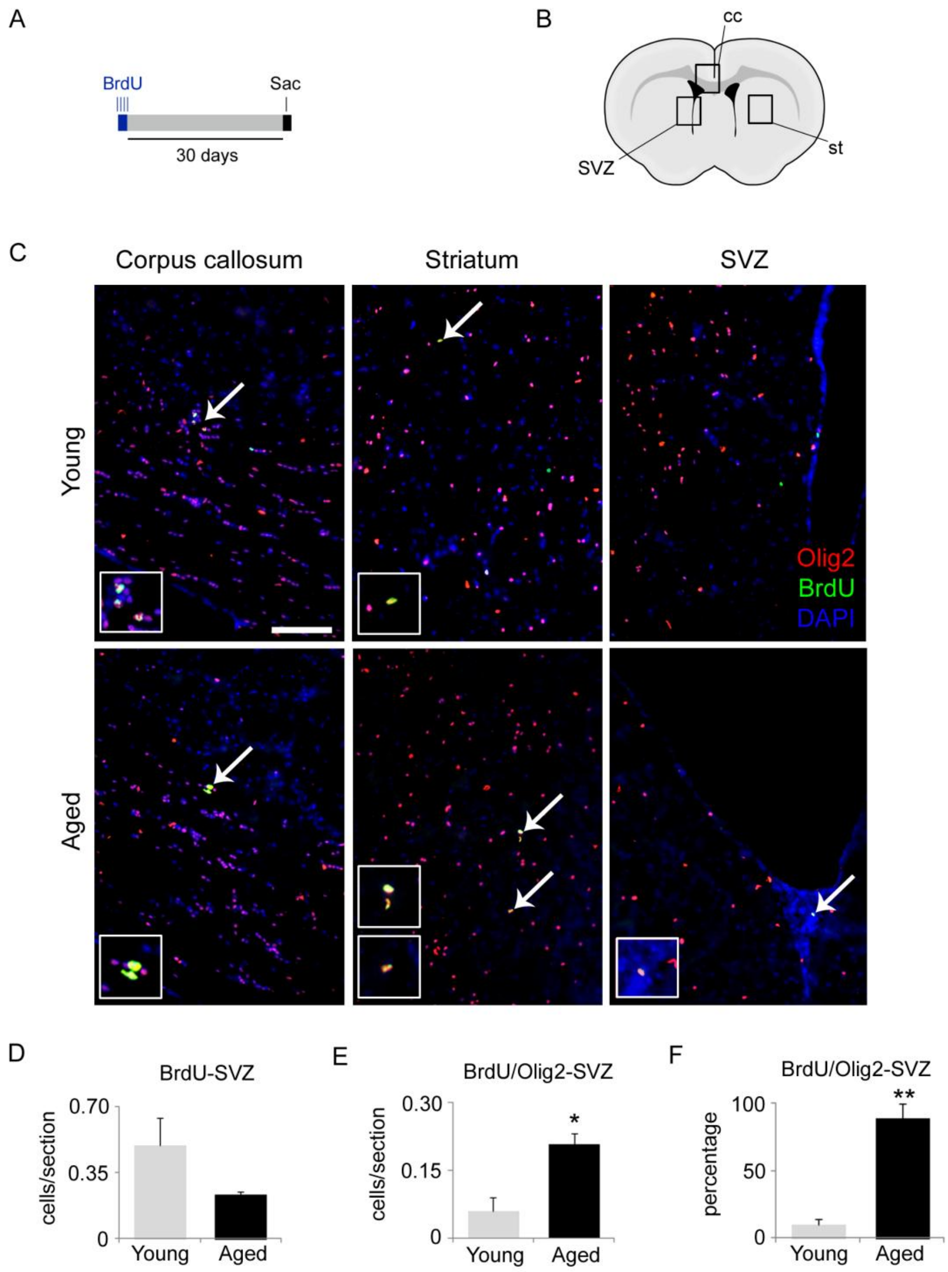

Fig. S6. The generation of the Olig2 lineage is maintained in the aged SVZ. (**A**) The animals received 4 doses of BrdU, separated by 2 hours, and were euthanized after 30 days. (**B**) Coronal section of the brain showing the studied areas in C. (**C**) Immunostaining against Olig2 (red) and BrdU (green) markers showed the presence of double positive cells (arrows) in the young and aged brain. (**D**) Bar graph depicting the number of BrdU+ cells in the aged SVZ, 30 days after treatment. (**E**) Bar graph depicting the number of BrdU+/Olig2+ cells in the aged SVZ, 30 days after treatment. Note the increase of new Olig2+ cells in the aged SVZ. (**F**) Bar graph depicting the percentage of Olig2+ cells co-expressing BrdU marker. Note the increase in the proportion of newly generated oligodendrocytes in the aged SVZ. Cc, corpus callosum; sac, sacrifice; st, striatum. Scale bar: C 100 µm. *p<0.05, **p<0.01

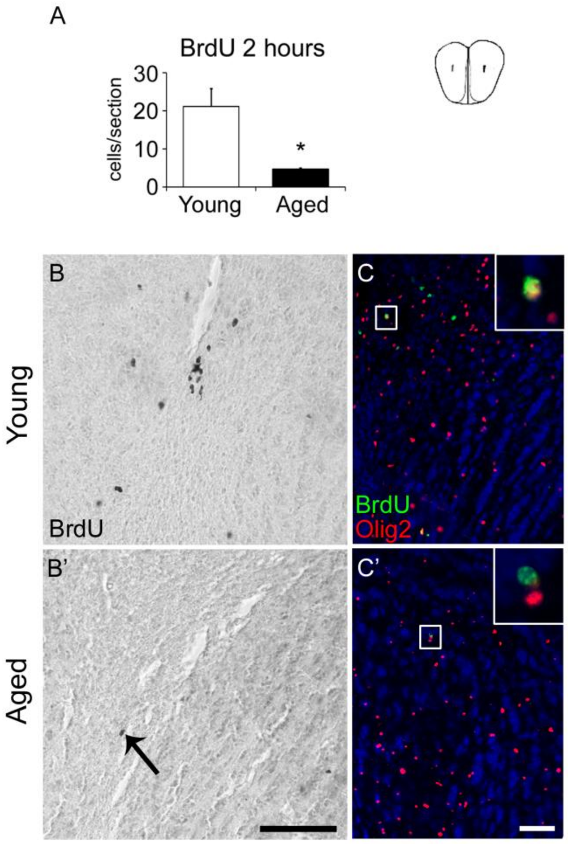

Fig. S7. Endogenous proliferating cells in the OB were decreased in the aged brain. (**A**) Bar graph depicting the number of BrdU+ cells in animals that were injected with a single dose of BrdU and euthanized 2 hours after. Note the significant reduction in the aged brain. (**B**) Immunostaining against BrdU in OB coronal sections representing bar graph in A. (**C**) Immunostaining against BrdU (green) and Olig2 (red) in OB coronal sections revealing a decrease of both markers during aging. Note the co-expression of both markers in the young OB, but not in the aged mice. Scale bar: B 10 µm, C 50 µm.

Table S1. Primary antibodies used in this study

Antibody	Species, type	Dilution	Antigen	Cat. number, manufacturer	Specificity
BrdU	mouse monoclonal, IgG	1:150	Bromodeoxyuridine conjugated to bovine serum albumin.	MO744, Dako (Glostrup, Denmark)	Cells in S-phase
BrdU	rat monoclonal, IgG	1:200	The details of the antigen for this antibody are not available	AB6326, Abcam (Cambridge, MA, USA)	Cells in S-phase
Dcx	goat polyclonal, IgG	1:200	Peptide mapping at the C-terminus of Dcx of human origin.	SC-8066, Santa Cruz Biotechnology (Santa Cruz, CA, USA)	Young neurons
GFAP	mouse monoclonal, IgG	1:500	Purified GFAP from porcine spinal cord	MAB360, Millipore (Billerica, MA, USA)	Astrocyte
GFAP	rabbit polyclonal, IgG	1:500	GFAP isolated from cow spinal cord.	Z0334 Dako (Glostrup, Denmark)	Astrocyte
Ki67	Rabbit monoclonal, IgG	1:200	A synthetic peptide derived from the human Ki67 ptotein.	RM-9106, Thermo Scientific (Fremont, CA, USA)	Cells in late G1-, S-, M-, and G2-phases
NeuN	Mouse monoclonal, IgG	1:700	The details of the antigen for this antibody are not available	MAB377, Millipore (Billerica, MA, USA)	Mature neurons
Olig2	Rabbit polycoclonal, IgG	1:500	The details of the antigen for this antibody are not available	AB9610, Millipore (Billerica, MA, USA)	Oligodendroglia cells
